# Supplementary material for: Hyperacidification of Citrus fruits by a vacuolar proton-pumping P-ATPase complex
Source: Nat Commun. 2019 Feb 26;10:744. doi: 10.1038/s41467-019-08516-3 (PMC6391481; doi:10.1038/s41467-019-08516-3)
Supplement: Supplementary file 1 — Supplementary Information [file 41467_2019_8516_MOESM1_ESM.pdf]

Supplementary Information

Hyperacidification of *Citrus* fruits by a vacuolar proton-pumping P-ATPase complex

Strazzer et al.

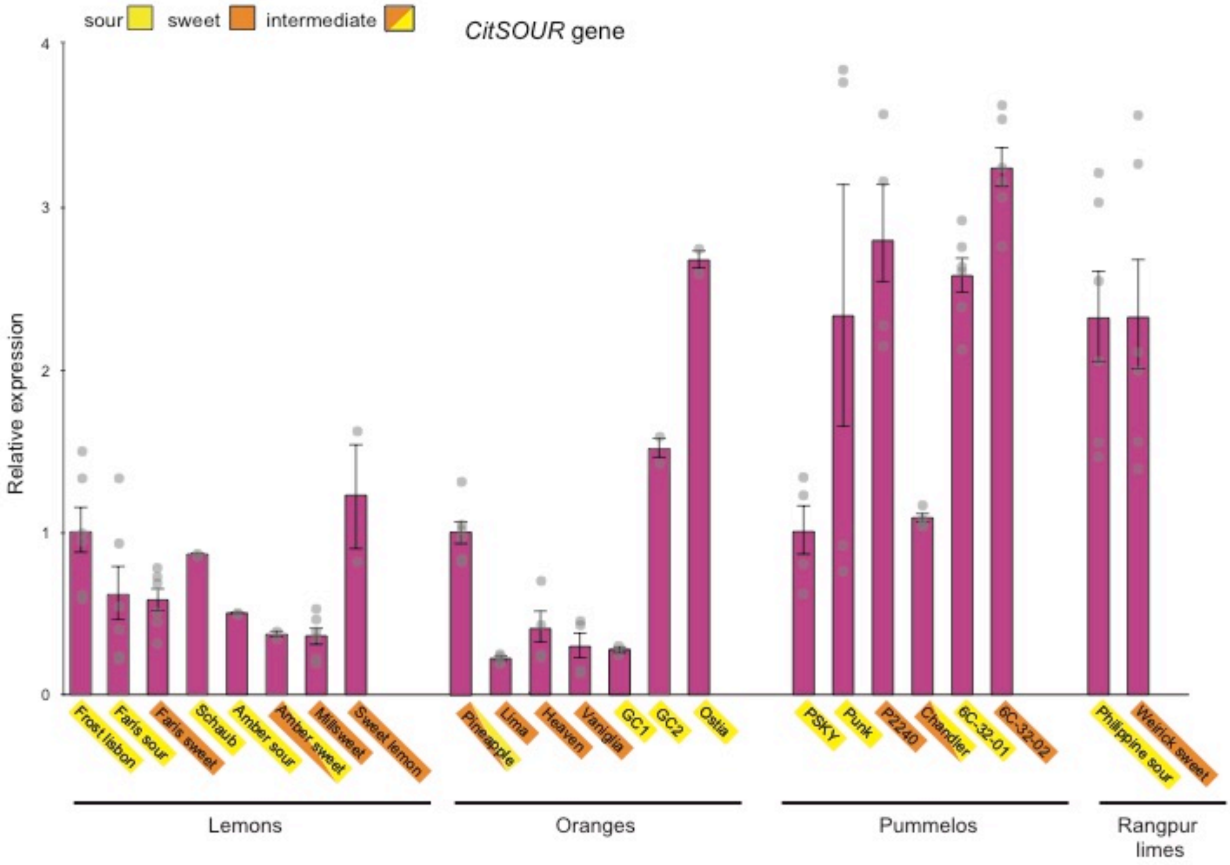

Supplementary Figure 1. Expression of *CitSOUR* in juice cells of various *Citrus* fruits.

Relative gene *CitSO* expression levels in juice cells were determined by real-time RT-PCR. Four genes (*CitANKYRIN, CitANNEXIN2* and *CitRIBOSOMAL PROTEIN S10,* and *CitACTIN11*) served as a constitutively expressed reference mRNAs for normalization. Gene identifiers of *CitSO* homologs are given in Supplementary Table 2. Values shown are mean ± SE; *n* = number of samples from different fruits X number of technical replicates of each. Source data are provided as a Source Data file.

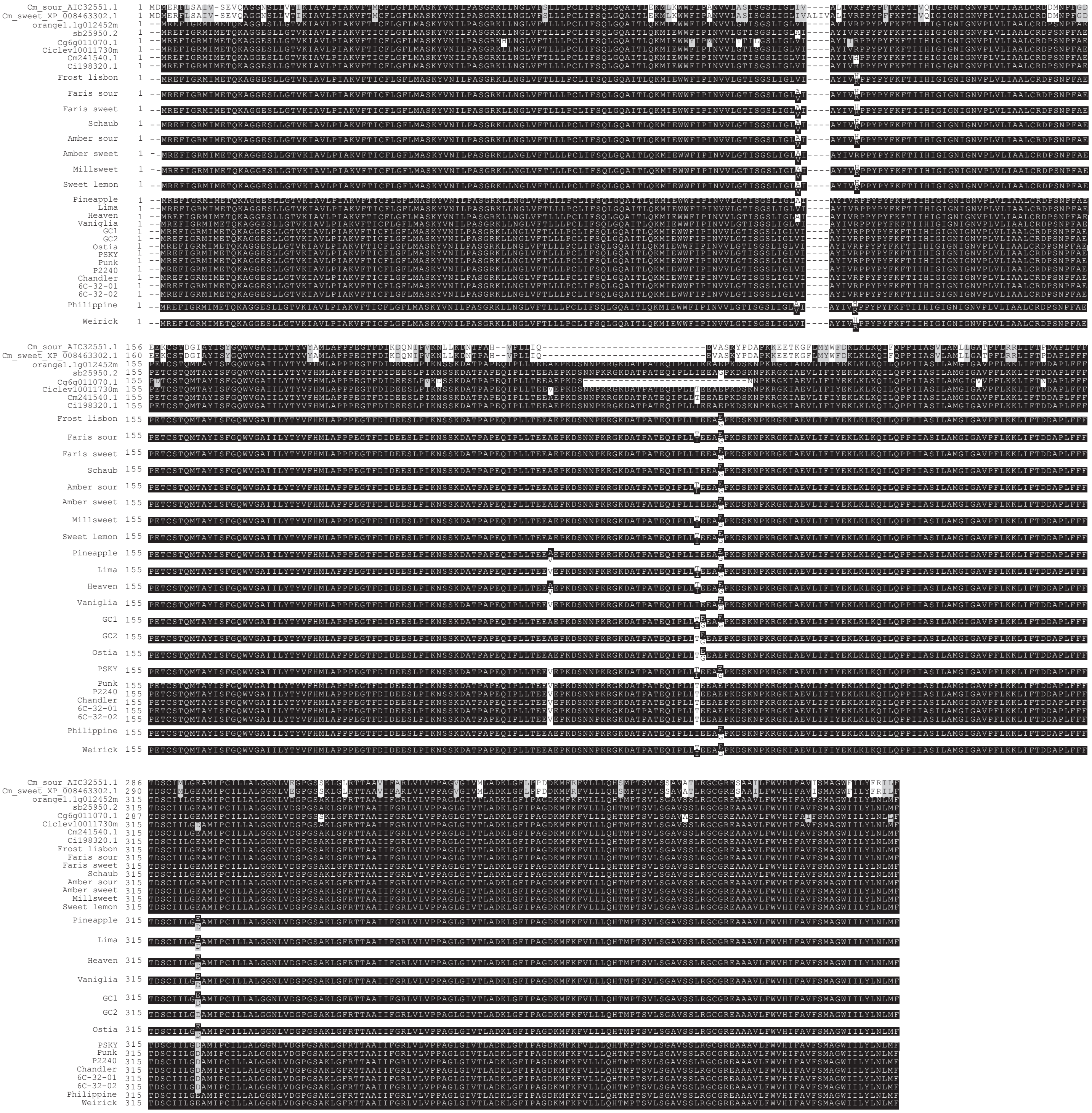

Supplementary Figure 2. Alignment of predicted CitSO proteins expressed in fruits from distinct varieties

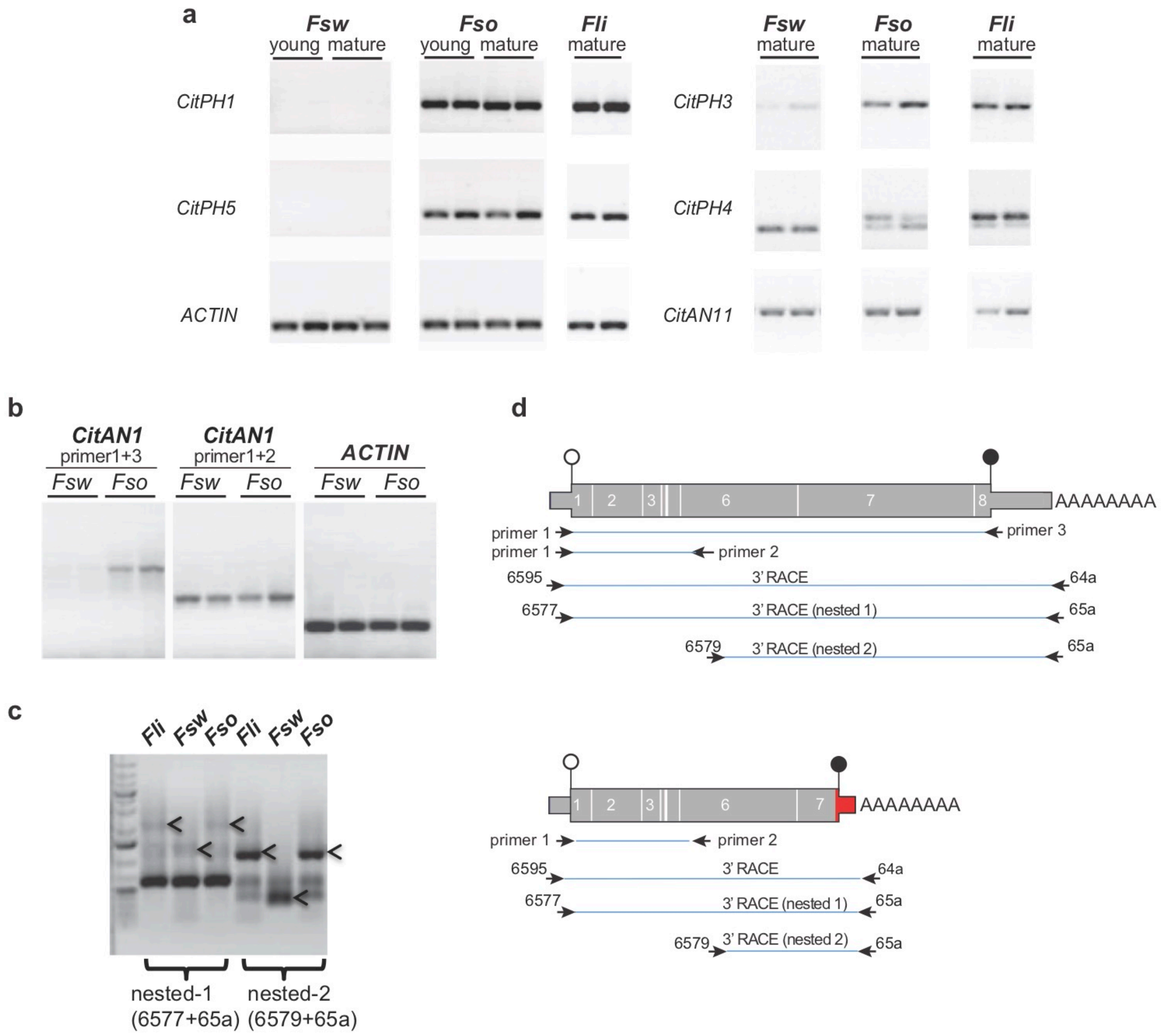

Supplementary Figure 3. Transcripts of *CitPH1*, *CitPH5* and the regulatory genes *CitAN1*, *CitPH3*, *CitPH4*, *CitAN11* in juice cells of *Frost Lisbon* (*Fli*) *Faris sour* (*Fso)* and *Faris sweet* (*Fsw*) lemons*.*

(**a**) RT-PCR analysis of *CitPH1*, *CitPH5*, *CitPH3*, *CitPH4*, *CitAN11* mRNAs in young and mature *Fli*, *Fso, Fsw* lemons. (**b**) RT-PCR analysis of *CitAN1* mRNA in *Fso* and *Fsw* lemon juice cells. (**c**) Identification of polyadenylation sites in truncated *CitAN1* mRNA by 3' RACE-PCR. **(*d*)** Diagram of functional *CitAN1* mRNA from *Fso* fruits (top) and truncated mRNA from *Fsw* fruits (bottom). Positions and orientation of the PCR primers used are indicated below the cDNA diagrams. Sequences of primers used are shown in Supplementary Table 3.

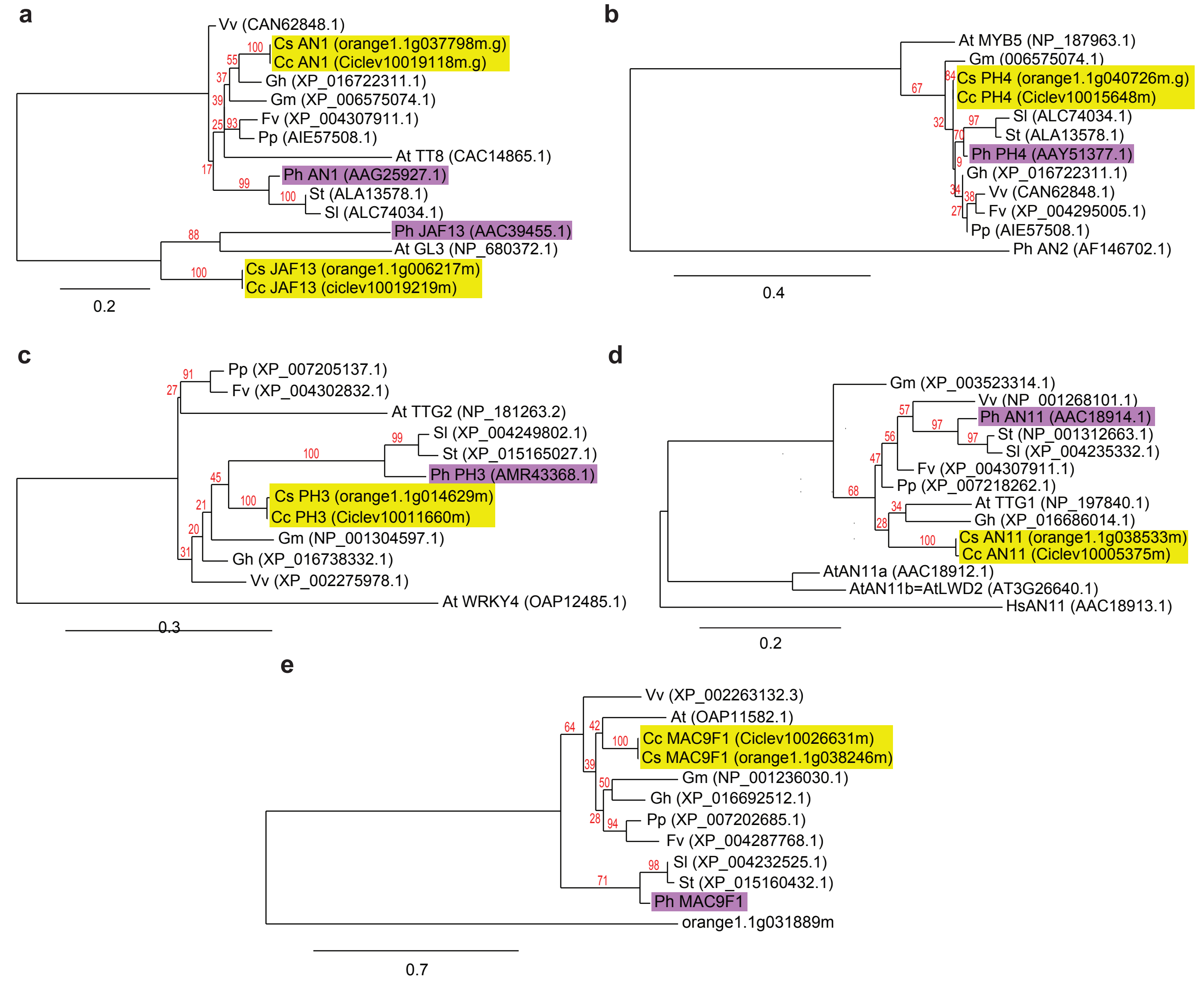

Supplementary Figure 4. Identification of *AN1, PH4*, *PH3*, *AN11* and *MAC9F1* homologs from *Citrus*.

(**a**) Phylogenetic analysis of AN1 homologs. The closely related HLH proteins JAF13 from petunia and GLABROUS3 (GL3) from Arabidopsis served as an outgroup. (**b**) Phylogenetic analysis of PH4 homologs. The closely related MYB protein ANTHOCYANIN2 (AN2) from petunia was used as an outgroup. (**c**) Phylogenetic analysis of PH3 homologs. The next most similar WRKY protein from Arabidopsis (WRKY4) was used as an outgroup. Additional evidence for orthology of PH3, TTG1 and *Citrus* homologs can be found elsewhere[1]. (**d**) Phylogenetic analysis of AN11 homologs. The Arabidopsis homolog is TRANSPARENT TESTA GLABRA1 (TTG1). Two TTG1 paralogs, initially named AtAN11a and AtAN11b, and the human homolog HsAN11 served as an outgroup[2]. (**e**) Phylogenetic analysis of MAC9F1 homologs. MAC9F1 is encoded by a single gene in all species analyzed and not part of a family. As an outgroup we used the next most similar protein from *C. sinensis* (orange 1.1g031889).
For each protein a Genbank accession number and/or gene identifier is given in brackets. Branch support is calculated on the basis of 300 bootstraps and indicated as percentage. Prefixes denote the species of origin as follows: At, *Arabidopsis thaliana*; Cs, *Citrus sinensis*; Cc, *Citrus clementina*; Fv, *Fragaria vesca*; Gh, *Gossypium hirsitum*; Gm, *Glycine max*; Hs, *Homo sapiens*; Ph, *Petunia hybrida*; Pp, *Prunus persica*; St, *Solanum tuberosum*; Sl, *Solanum lycopersicum*; Vv, *Vitis vinifera*. Gene IDs of homologs in other *Citrus* species/varieties are given in Supplementary Table 2.

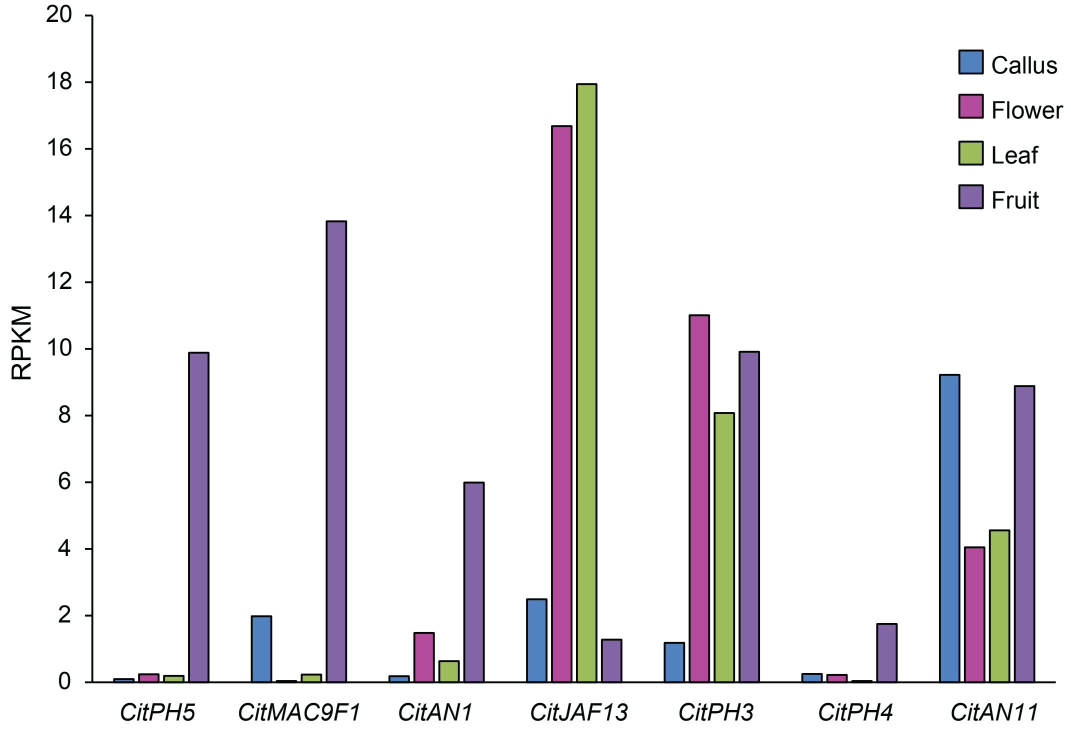

Supplementary Figure 5. Expression pattern of Citrus homologs of *PH5*, *MAC9F1*, *AN1*, *JAF13*, *PH3*, PH4 and AN11.

Expression data were extracted from publicly available genome-wide RNAseq data[3].

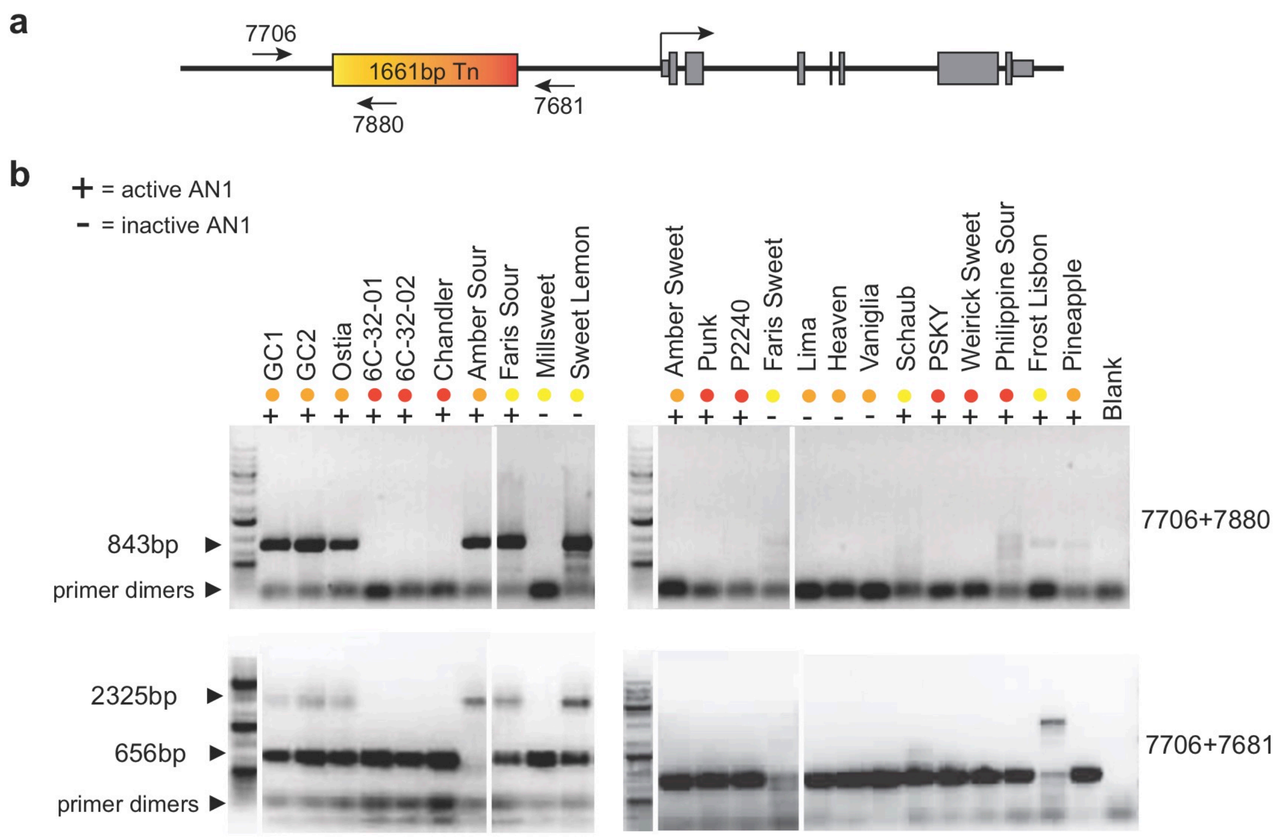

Supplementary Figure 6. PCR analysis of *CitAN1* promoter in different *Citrus* varieties.

(**a**) Diagram showing the structure of a *CitAN1* allele with an insertion of a 1661-bp *hAT*-type transposon (Tn; yellow/orange rectangle) in the promoter. (**b**) PCR analysis to detect the presence of transposon copies in the promoter of *CitAN1* alleles from different *Citrus* fruits.

Yellow circles indicate lemon varieties, orange circles indicate sweet and sour orange varieties and red circles indicate pummelo varieties. Note that PCR with primers 7706 and 7880 only yields a PCR product when the transposon is present in the fragment, while the size of the fragment indicates its position (top panels). PCR with primers 7706 and 7681 yields PCR products of two sizes when the transposon is heterozygous. Primer sequences used are shown in Supplementary Table 5-6.

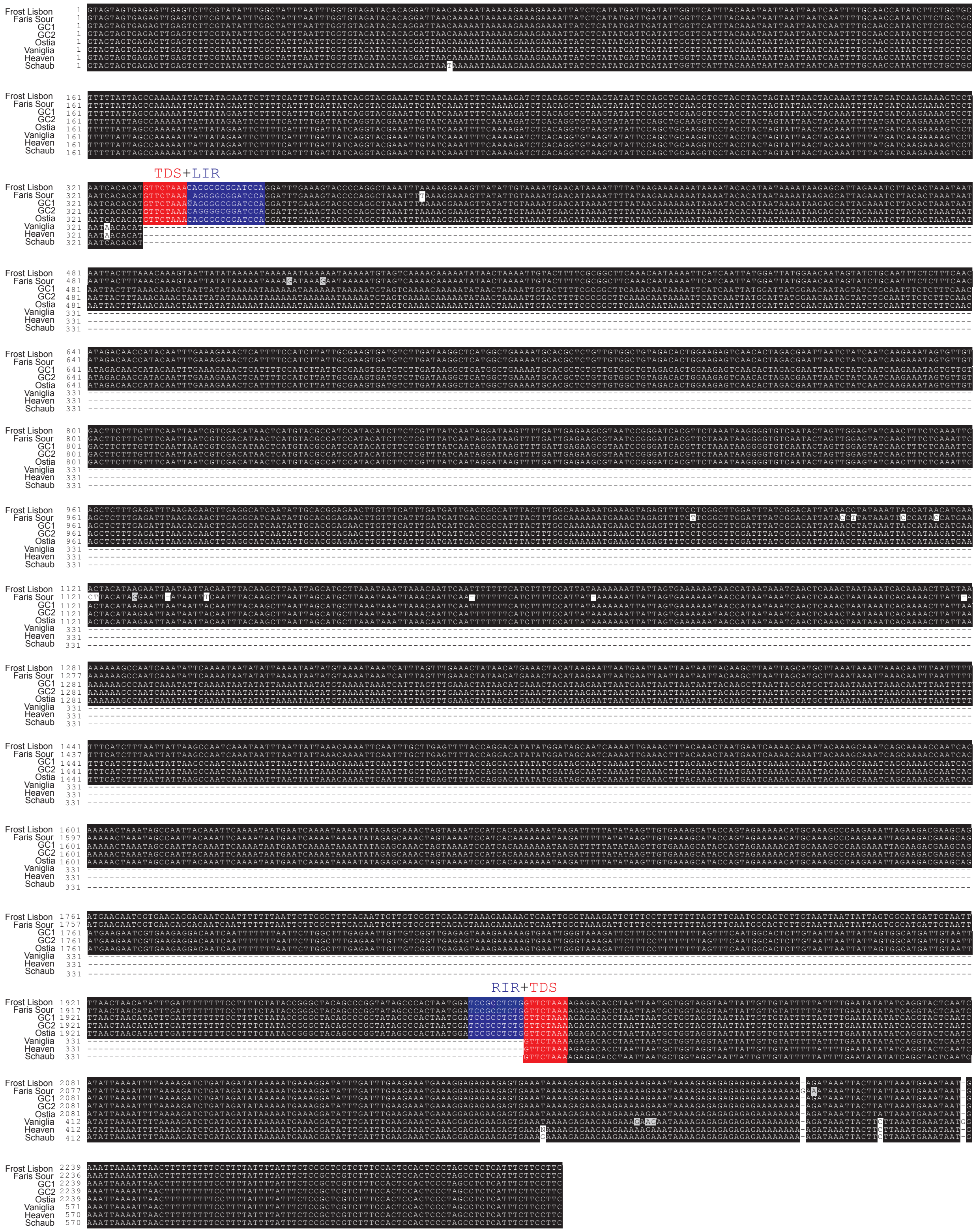

Supplementary Figure 7. Analysis of AN1 promoters

Alignment of AN1 promoter sequences of 'Frost Lisbon', 'Faris' sour, 'Schaub', GC1, GC2, Ostia, Vaniglia, Heaven (primers 7706 and 7681) The left and right inverted repeats (LIR, RIR) are marked in blue. The 8-bp target site duplication (TSD) caused by the insertion is marked in red.

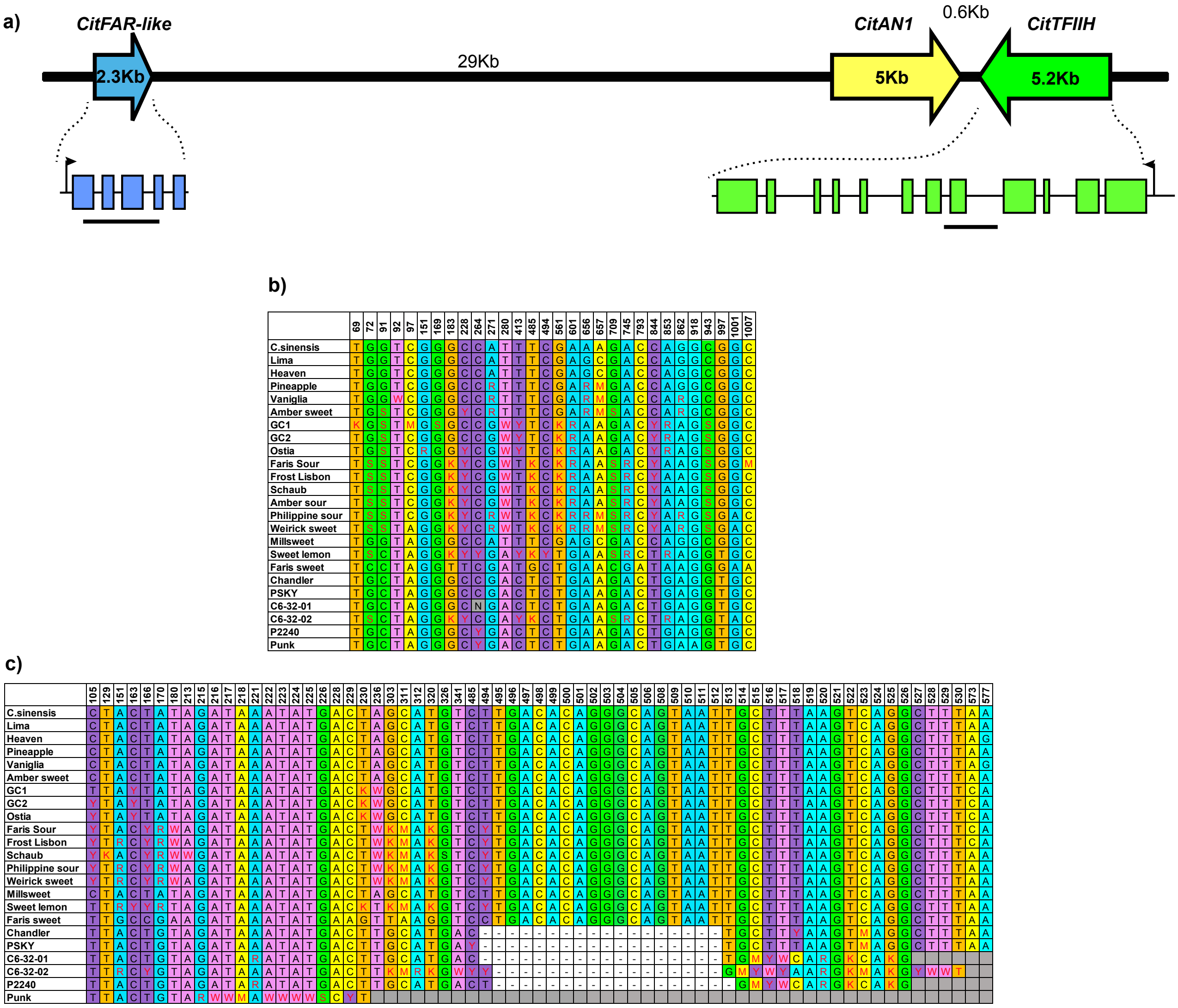

Supplementary Figure 8. Polymorphisms in *CitFAR-like* and *CitTFIIH.*

(**a**) Diagram showing structure of the genomic region surrounding *CitAN1* in *Citrus sinensis* cv Pineapple Ridge (top). The intron-exon structure *CitFAR-like* (orange1.1g014509) and *CitTFIIH-like* (orange 1.1g004358m) is shown below; the black line bar indicates the region (re)sequenced to establish homo- or heterozygosity of this genomic region.
(**b**) Distribution of SNPs found in the (re)sequenced region (~ 1 kb) of *CitFAR*-like. (**c**). Distribution of SNPs found in (re)sequenced region (~0.6 kb) from the *CitTFIIH-like*.
Nucleotide ambiguity codes indicating mixed sequences due to heterozygosis or low quality sequence reads are indicated in red letters, following IUPAC code (W = A/T; S=G/C; M= A/C; K=G/T; R=A/G; Y=C/T). Dashes on a white background indicate absence of nucleotides (indel). Grey shading indicates sequences not determined.
Primer sequences used for PCR amplification and sequencing are described in Supplementary Table 4.

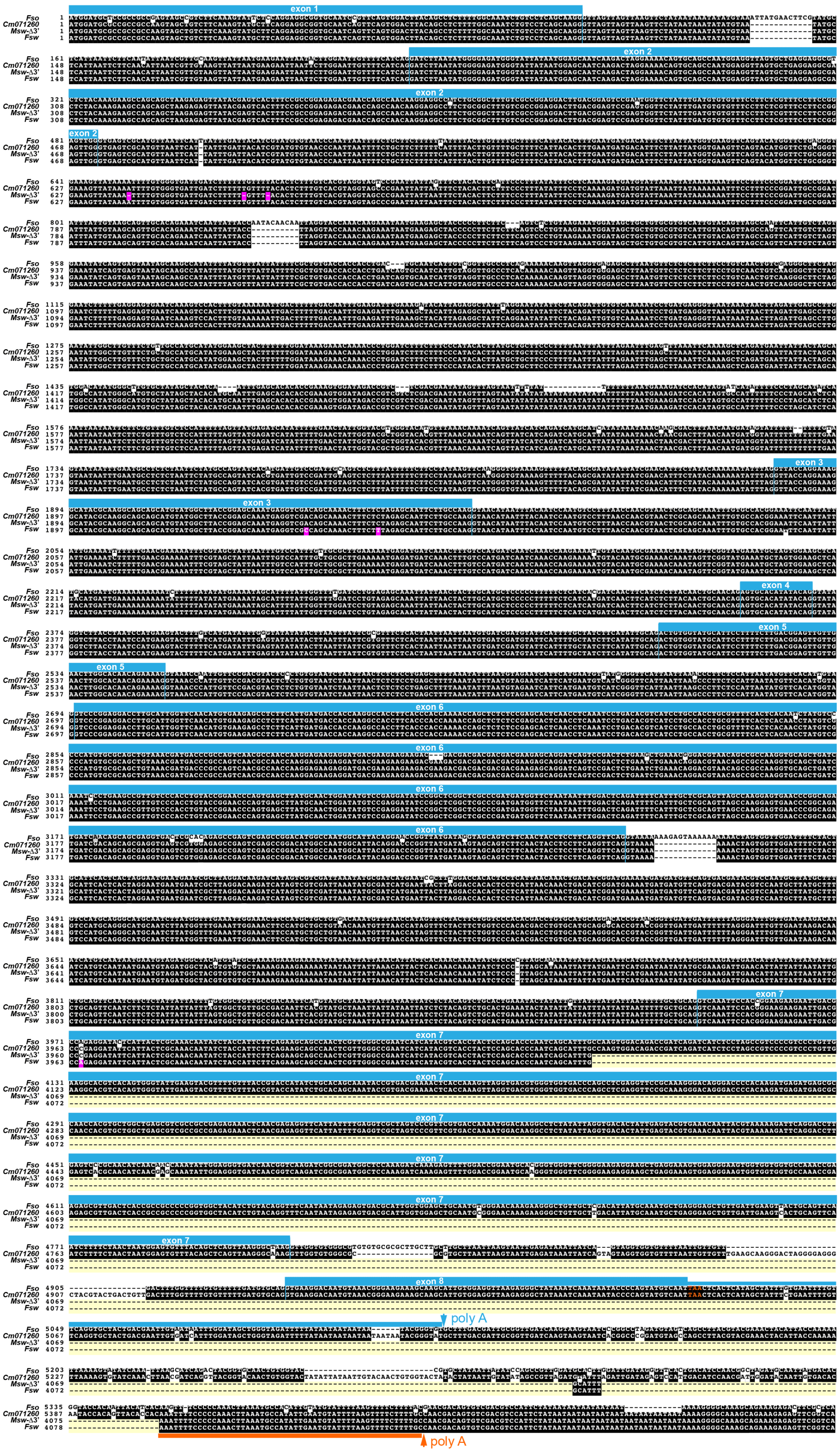

Supplementary Figure 9. Comparison of the *CitAN1* alleles from 'Faris' sour (*Fso*), 'Faris' sweet (*Fsw*) and 'Millsweet' lemons (*Msw*) and *C. medica*.

Blue bars overlying the sequences indicate exons. The deletion in *citan1*^{Fsw} and *citan1*^{Msw} is indicated by yellow shading, the downstream sequence until the polyadenylation site is indicated with an orange bar below the sequence. SNPs distinguishing *citan1*^{Fsw} and *citan1*^{Msw} are marked by magenta shading.

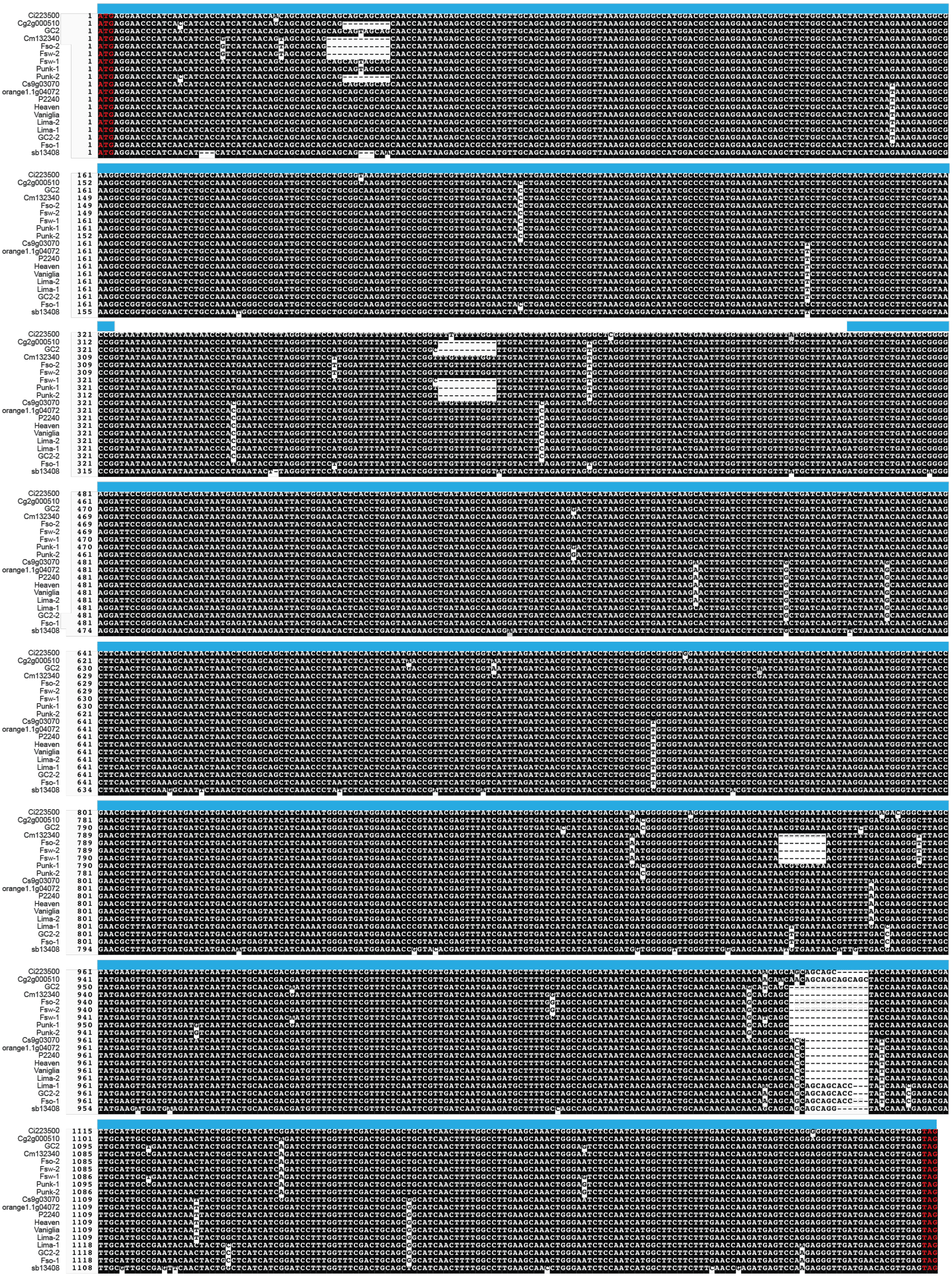

Supplementary Figure 10. Alignment of *CitPH4* alleles from *Citrus* species and varieties.

Blue bars overlying the sequences indicate exons. Start and stopcodons are indicated by red lettering.

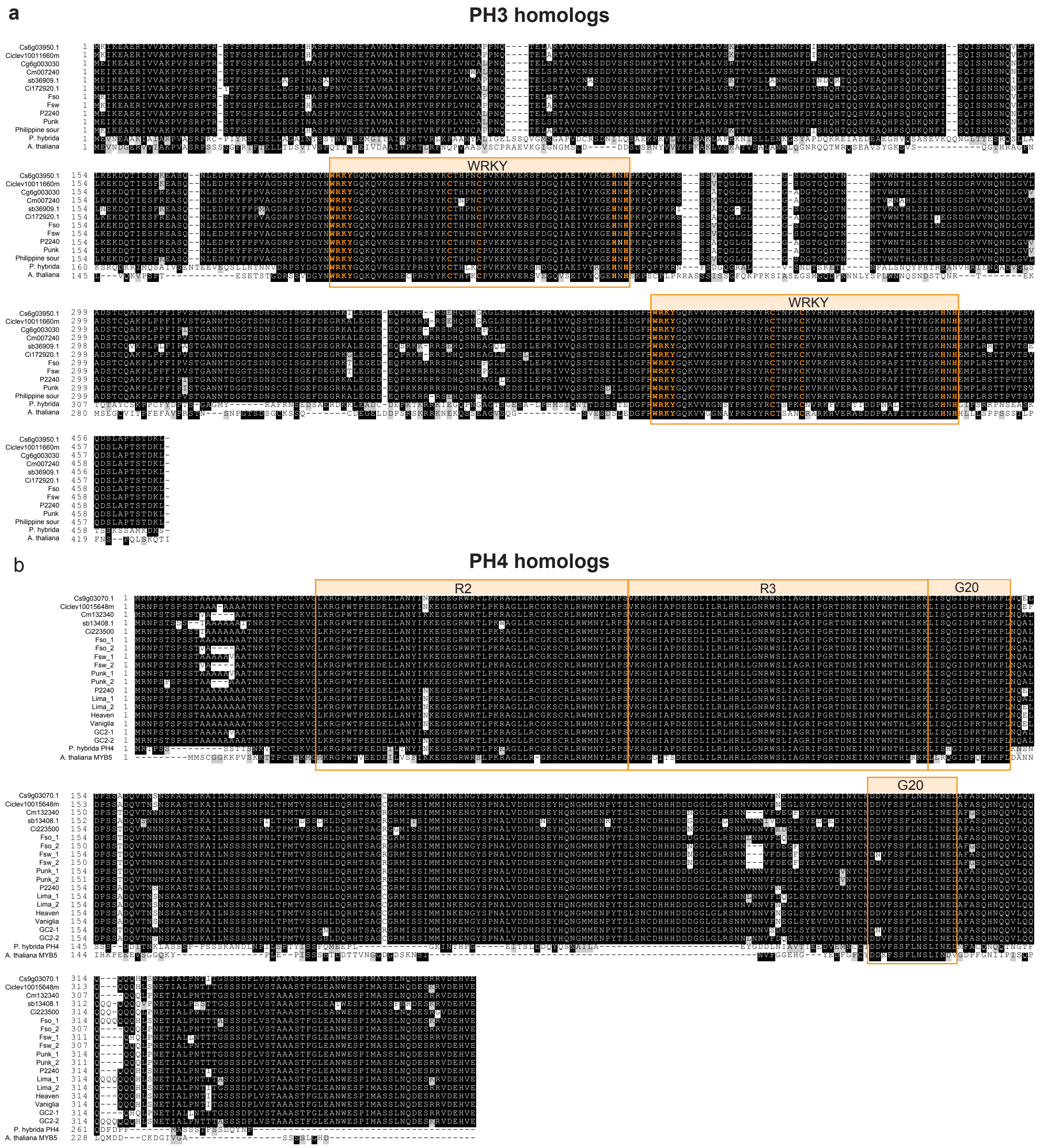

Supplementary Figure 11. Comparison of CitPH3 and CitPH4 proteins

(**a**) Alignment of CitPH3 proteins from different *Citrus* varieties with PH3 from petunia and the functionally interchangeable homolog TTG2 from *Arabidopsis*. The two WRKY domains are marked in orange. (**b**) Alignment of CitPH4 proteins from different *Citrus* accessions with PH4 from Petunia and its apparent homolog, AtMYB5, from Arabidopsis. The R2 and R3 repeats, which make up the MYB DNA binding domain and G20 boxes, which are signatures for this subclass of MYB proteins [4], are marked in orange.

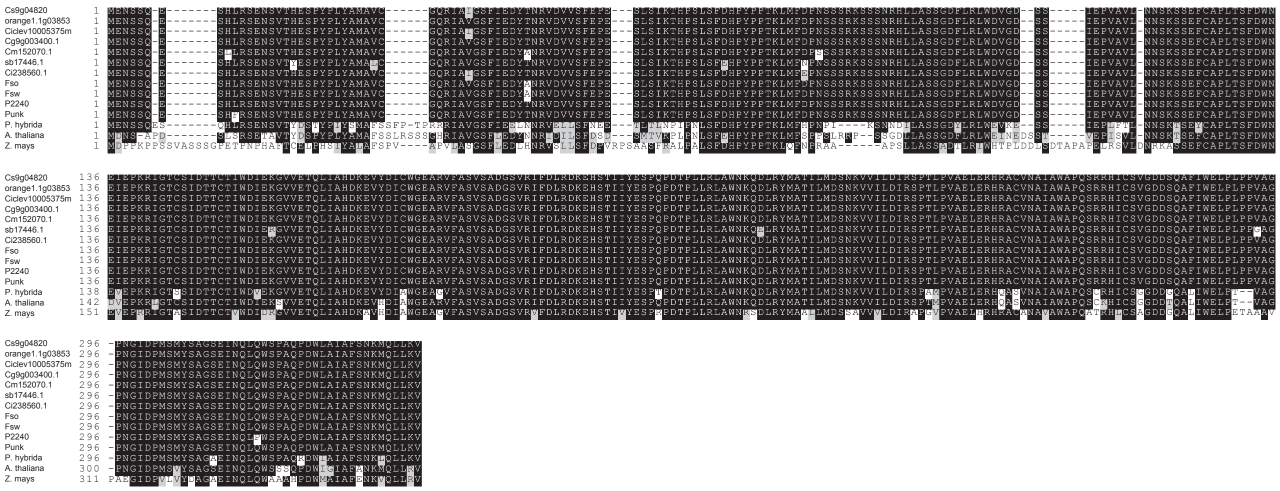

Supplementary Figure 12. Comparison of CitAN11 proteins

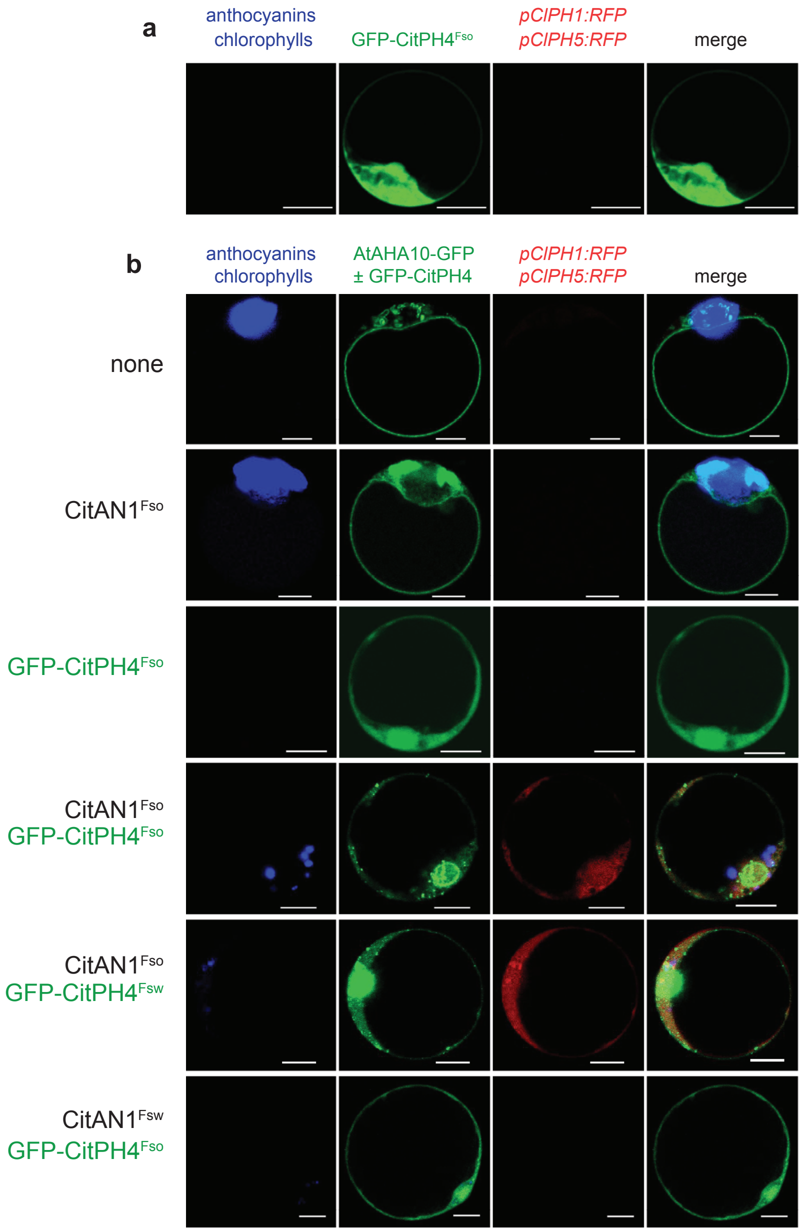

Supplementary Figure 13. Confocal micrographs of petunia *ph4* mesophyll petal protoplasts from stage 6 flowers 24 hrs after transformation *35S:GFP-CitPH4*^{Fso}, *35S:GFP-CitPH4*^{Fsw}, *35S:CitAN1*^{Fso}, *35S:CitAN1*^{Fsw}, *pCitPH1:RFP*, *pCitPH5:RFP* in different combinations.

(**a**) Petal mesophyll protoplast expressing *35S:GFP-CitPH4*^{Fsw} showing accumulation of GFP-CitPH4^{FSo} in nucleus and cytoplasm (**b**) Petal mesophyll protoplasts co-transformed with the reporter genes *pCitPH1:RFP*, *pCitPH5:RF,* the control gene *35S:AtAHA10-GFP* and different combinations of effector genes expressing *Fso* and *Fsw* alleles of CitAN1 and/or GFP-CitPH4, as indicated on the left. Note that the central vacuole lacks anthocyanins, indicating that these protoplasts originate from the petal mesophyll. Expression data on epidermal cells, which contain anthocyanins, are shown in Fig 2.
Size bars equal 10 µm.

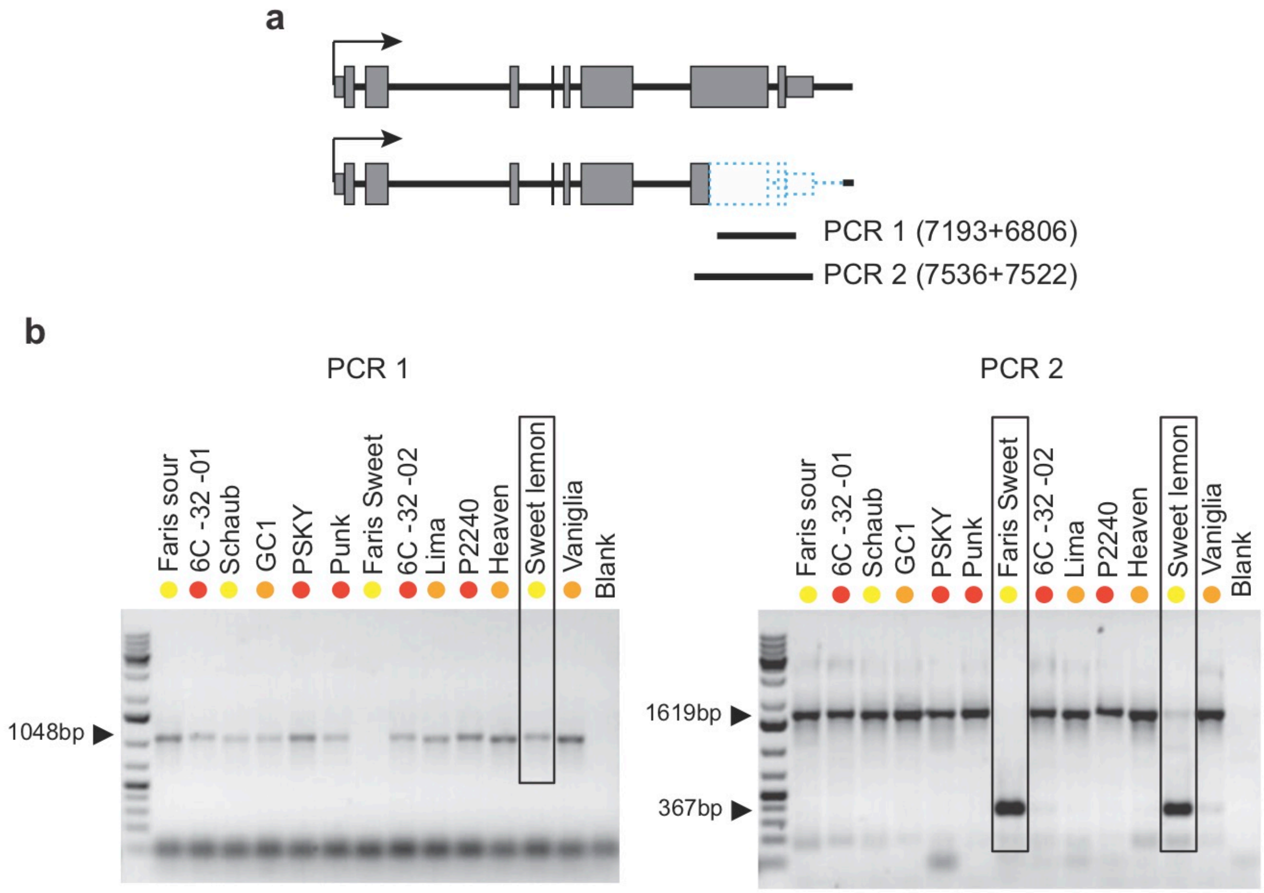

Supplementary Figure 14. Identification of truncated *CitAN1* allele in *Sweet lemon*.

(**a**) Diagram showing the structure of the functional *CitAN1* allele (top) and the mutant *citan1*^{Fsw} in which the 3'end of the gene is deleted (blue dotted lines). (**b**) PCR analysis of genomic DNA obtained from the fruits of different Citrus varieties with primers 7193 and 6806 (PCR1: this gives 1048-bp product when *CitAN1* is full size) and primers 7536 and 7522 (PCR2: this yield either 1619 bp in a full size allele or a smaller PCR product when the allele is truncated). Yellow circles indicate lemons, orange circles indicate sweet and sour oranges and red circles indicate pummelos. Primer sequences given in Supplementary Table 3. Source data are provided as a Source Data file.

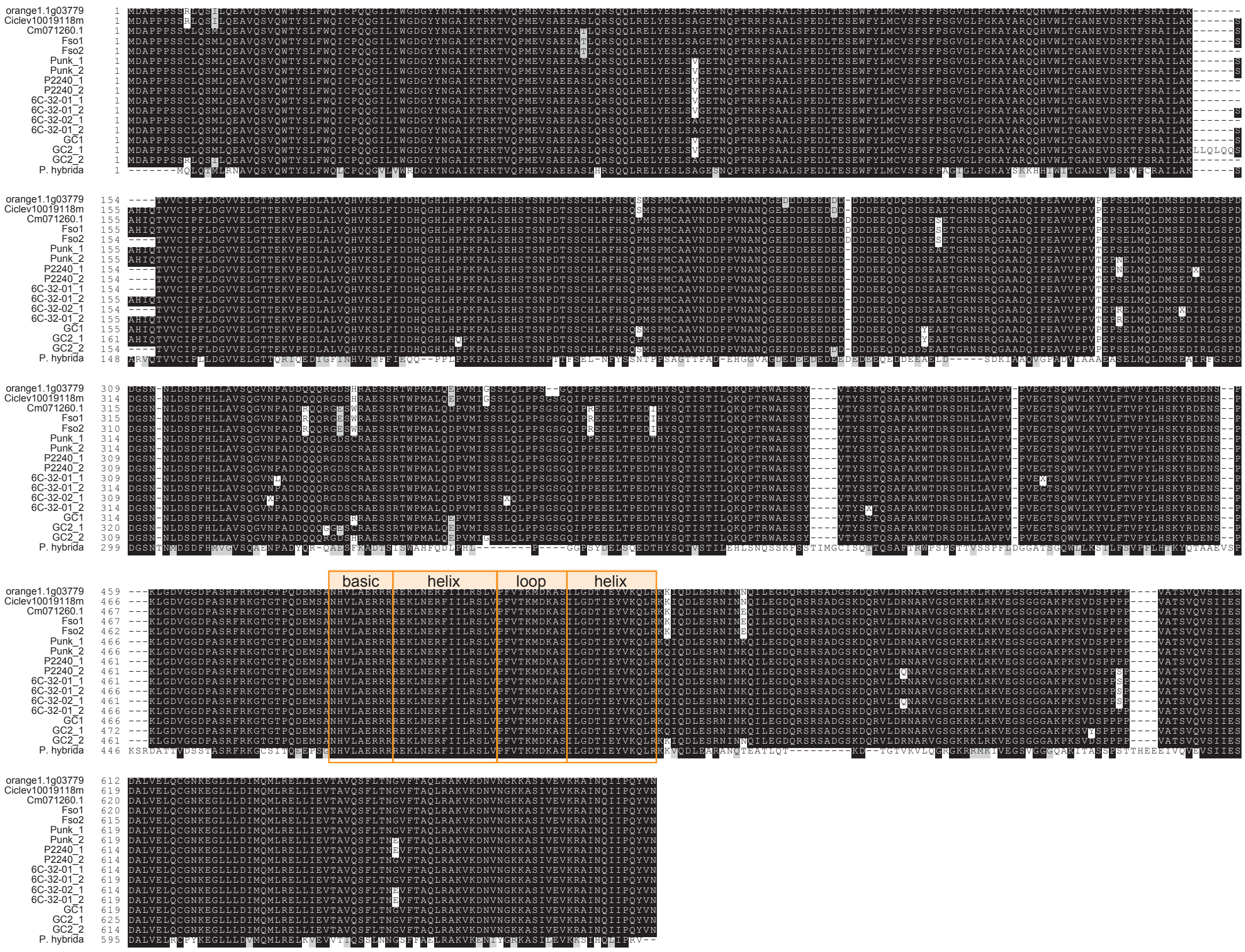

Supplementary Figure 15. Comparison of CitAN1 proteins encoded by distinct *Citrus* alleles.

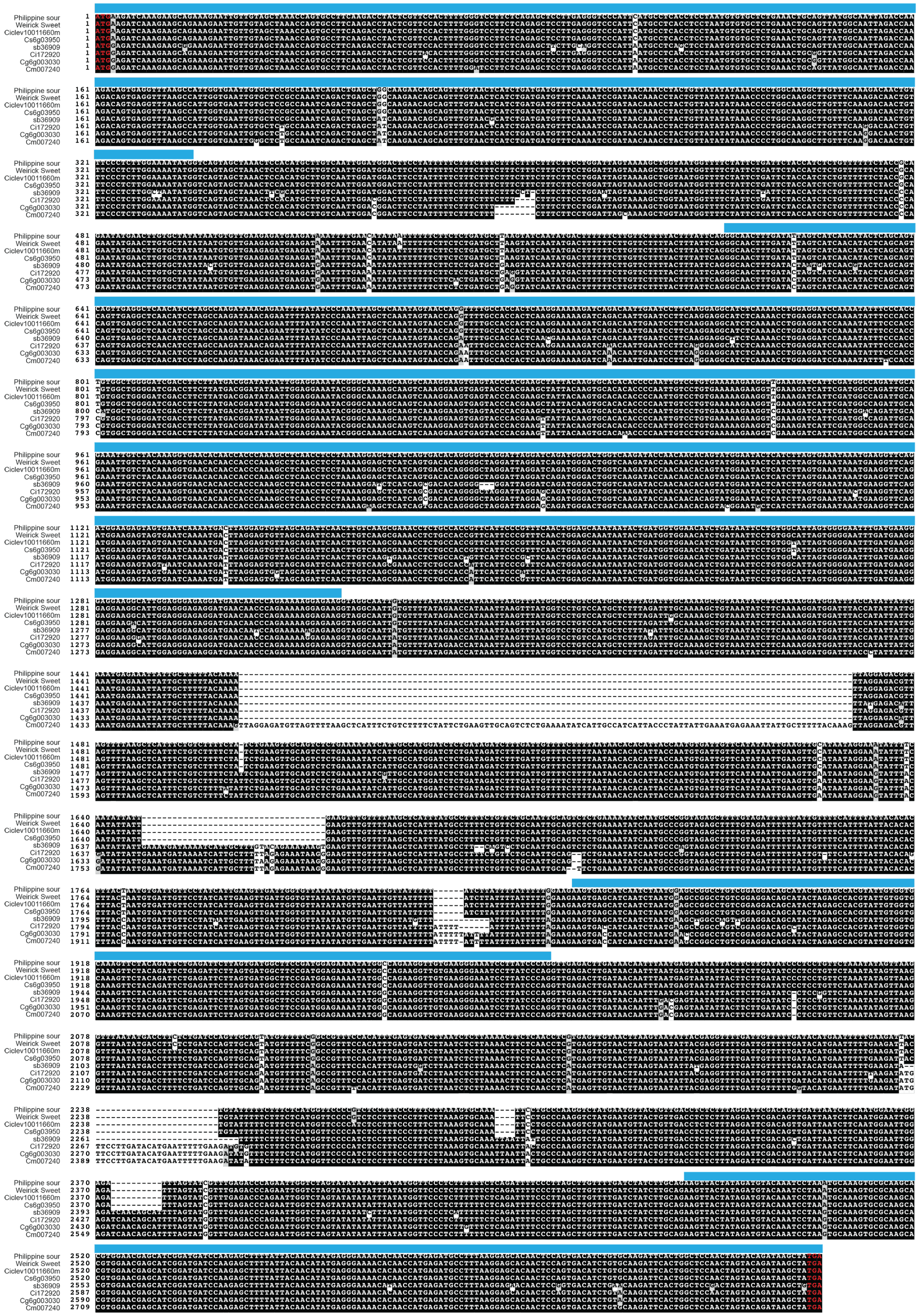

Supplementary Figure 16. Comparison of *CitPH3* alleles .

Blue bars overlying the sequences indicate exons. Red lettering indicates start and stop codon.

Supplementary Table 1. Description of *Citrus* varieties investigated in the study

Variety	Species/Origin	General Description	Taste	CRC[1]
Schaub rough lemon	*C. jambhiri*	Standard rough lemon	Sour	3879
Frost Lisbon lemon	*C.limon*	Standard commercial lemon	Sour	3176
Amber lemon Eureka	*C.limon*	Chimera. Trees have branches with purple flowers and yellow flesh and branches with white flowers and orange flesh.	Yellow flesh-fruits are sour like a standard lemon, orange flesh-fruits are sweet.	2429
Faris lemon	*C.limon*	Chimera. Trees have branches with purple flowers and branches with white flowers. Both fruits have yellow flesh.	Branches with purple flowers have acid tasting fruits, Branches with white flowers have sweet, almost acidless fruits.	2695
Millsweet limetta	*C.limetta*	It is the closest genotype to the sweet portion of Faris	Sweet, but with a relatively low pH	569
Unnamed sweet lemon	*C.limettioides?*	Sweet lime -type	Slightly acidic like limetta	3093
Pin Shan Kong Yau (PSKY)	*C.maxima*	Standard pummelo	Acid	2348
Siamese acidless pummelo P2240	*C.maxima*	Commonly used in breeding to reduce acid in progeny	Sweet, acidless	2240
Pummelo unknown (Punk)	*C.maxima*	White flesh, yellow rind	Sour (mildly acidic)	none
Chandler	*C.maxima* (hybrid of P2240×P2241)	The flesh varies in color from light pink to very dark pink	Sweet, but with a relatively low pH	3224
6C-32-01	*[Chandler*×(Weirick sweet rangpur lime ×*P2240*)]	One progeny tree from a cross that segregates for acidity	Sour	none
6C-32-02	*[Chandler*×*(* Weirick sweet rangpur lime ×*P2240)]*	One progeny tree from a cross that segregates for acidity	Sweet	none
Weirick sweet rangpur lime	*C.limonia*	It is the male parent of record for the male hybrid parent of 6C-32-01 and 6C-32-02	Sweet	1684
Philippine rangpur lime	*C.limonia*	Standard rangpur type. Results on same Affymetrix SNP chip as Weirick were nearly identical	Sour	2318
Pineapple orange	*C.sinensis*	Standard seedy orange	Sweet, but with a relatively low pH	3858
Lima orange	*C.sinensis*	Low acid type of orange	Sweet, taste almost acidless	950
Orange of Heaven	*C.sinensis*	Low acid type of orange	Sweet	371
Vaniglia Sanguigno	*C.sinensis*	Low acid type of orange. Pink flesh pigmented by lycopene.	Sweet	3801
unnamed sour orange (GC1)	*C.aurantium*	Fruit from tree in the botanical garden *Jardin Botanico* Canario Viera y Clavijo, in Gran Canaria (Spain). It is a normal orange type.	Sour	none
unnamed sour orange (GC2)	*C.aurantium*	Fruit from tree in the town Agüimes, Gran Canaria (Spain). It is a more yellowish orange, smaller than an ordinary orange.	Sour	none
unnamed sour orange (Ostia)	*C.aurantium*	Fruit from a tree grown in Ostia (Italy)	Sour	none

[1] CRC= catalog number used by the Citrus Variety Collection

Supplementary Table 2. Gene identifiers of orthologous genes in distinct *Citrus* species and varieties.

gene	*C. sinensis* cv 'Valencia' Sweet orange	*C. sinensis* cv 'Ridge Pineapple' Sweet orange	*C. clementine* cv 'Clemenules' Mandarin	*C.maxima* Pummelo	*C.ichangensis* Papeda	*C.medica* Citron	*A.buxifolia* Chinese Box Orange
CitAN1	Cs5g31400	orange1.1g037798m	Ciclev10019118m	Cg5g035630	Ci208360<br>Ci296140	Cm071260	sb18483<br>sb18180
CitJAF13	orange1.1t00363	orange1.1g006217m<br>orange1.1g007527<br>orange1.1g007097	Ciclev10019219m<br>Ciclev10019338m<br>Ciclev10019401m	Cg5g042050	Ci001120	Cm035480	sb30723
CitAN11	Cs9g04820	orange1.1g038533m	Ciclev10005375m	Cg9g003390	Ci238560	Cm152070	sb17505
CitPH3	Cs6g03950	orange1.1g014629m	Ciclev10011660m	Cg6g003030	Ci172920	Cm007240	sb36909
CitPH4	Cs9g03070	orange1.1g040726m	Ciclev10015648m	Cg2g000510	Ci223500	Cm132340	sb13408
CitPH1	Cs1g20080	orange1.1g045750m	Ciclev10027273m	Cg1g008240	Ci023800	Cm203880	sb10432
CitPH5	Cs1g16150	orange1.1g002768m	Ciclev10024807<br>Ciclev10024879	Cg1g013430	Ci172390	Cm026870	sb29446
CitMAC9F1	Cs1g26060	orange1.1g038246m	Ciclev10026631m	Cg1g001120	Ci105200	Cm173840	sb16135
CitSO	Cs6g10480	orange1.1g013452m	Ciclev10011730m	Cg6g011070	Ci198320	Cm241540	sb25950
CitACTIN11	Cs7g29220	orange1.1g017081m	Ciclev10031847m	Cg7g004230	Ci071400	Cm161310	sb19856
CitANNEXIN2	Cs1g01540	orange1.1g021204m	Ciclev10027333m	Cg1g029460	Ci156610	Cm152920	sb23410
CitANKYRIN	orange1.1t02270	orange1.1g018578m	Ciclev10012463m	Cg6g012460	Ci194380	Cm119200	sb37601
CitRIBOSOMALPROTEIN S10	Cs9g19200	orange1.1g029742m	Ciclev10006024m	Cg9g027530	Ci055690	Cm027640	sb21187

Sequences were identified at and retrieved from Phytozome (https://phytozome.jgi.doe.gov/pz/portal.html) and *Citrus sinensis* Annotation Project (CAP)(http://citrus.hzau.edu.cn/orange/index.php) databases

Supplementary Table 3. Primers used for the amplification of *CitPH1*, *CitPH5*, *CitAN1*, *CitPH3*, *CitPH4*, *CitAN11* genes and/or cDNA

primer	gene	sequence 5'-3'	orientation
8932	*CitSO*	GAT TTG TGT ATC TCT CTT CAG AG	F
8933	*CitSO*	CTG CGA AGA AAA GCT TCC GAG	R
6524	*CitPH1*	ATG GGA GGA CTC AAA ATC TTC TC	F
4383	*CitPH1*	CCA GGA AAA AGG TCT CCA GGT TC	R
6530	*CitPH5*	GG AGC TCT GTT GAT GTG TGC A	F
6529	*CitPH5*	TCA GAC TGT GTG AGC TGC TTG	R
6538	*CitPH3*	TCA GAC TGT GTG AGC TGC TTG	F
6540	*CitPH3*	AGG ACG ACT ATA TCA GCC GTT	R
6611	*CitPH3*	ACC ACT GAA ATG TGA TCG GCA TTT CAT GCT	F
6612	*CitPH3*	TGT CAG GTA ATA TGT TGG CTC GA	R
6544	*CitPH4*	ATG AGG AAC CCA TCA ACA TCA C	F
6546	*CitPH4*	CTT GGC TTA TCA GCT TCT TAC TC	R
6573	*CitPH4*	GAG GTG GAA CAA GTG CAT GAA	R
6614	*CitAN11*	ATG GAA AAC TCA AGC CAA GAA TC	F
6615	*CitAN11*	AGT TAG CTA TCC CAA ACG AGC A	R
6595	*CitAN1*	AGA AGC AGA GAA TCT CTG GCG GTG A	F
6577	*CitAN1*	CGGT GAA TAC AGT CGG TAA AGG A	F
6623	*CitAN1*	ATG GAT GCT CCG CCG CCG A	F
6592	*CitAN1*	G AGT TAA TTG ACA TAC TGG GG	R
6593	*CitAN1*	G AAA ATT TAA ATG CCA AAT GC	R
7642	*CitAN1*	GAA TAC AGT CGG TAA AGG AAT GGA TG	F
7590	*CitAN1*	GTC AGA TTC GGA GGC AGT AG	R
6806	*CitAN1*	TCG TAC GTA AGG CTG ACT ACA TCT	R
7193	*CitAN1*	GTC ACA GTG GGT ATT GAA GTA CGT	F
7522	*CitAN1*	GAG ATA AAG ACG TGT CAG ATT CGG A	R
7536	*CitAN1*	GGA AGA AGA ATT GAC GCC AGA G	F
64a	*attB2-oligo dT adaptor*	GAGACTGGACCACTTTGTACA	R
65a	*attB2-oligo dT adaptor*	GGGGACCACTTTGTACAAGAAAG	R

Supplementary Table 4. Primers used for the amplification of *AN1* flanking sequences

gene		sequence 5'-3'	orientation
8063	*CitTFIIH-like*	GGA AAC ATT CGC AGA GCT GAG	F
8065	*CitTFIIH-like*	GGT ACT CAC TGG AAG TTG GTC	R
8066	*CitFAR-like*	GAG GAT GTG GCT CCT AAA GAG A	F
8068	*CitFAR-like*	GAA TGG AGT ACT GAC TTA CAA GAG	R

Supplementary Table 5. Primers used for the amplification of *AN1* promoters

primer		sequence 5'-3'	orientation
7706	*CitAN1*	TGT AGT AGT GAG AGT TGA GTC TTC	F
7681	*CitAN1*	GAA GGA AGA AAT GAG AGG CTA G	R
7880	*CitAN1* (Tn-insertion)	CGT ACA TGA GTT ATG TCG ACG A	R

Supplementary Table 6. Primers used for the sequencing of *AN1* promoters

primer		sequence 5'-3'	orientation
7674	*CitAN1*	ACA GCA AAC AGC AGT AGG ACA G	F
7473	*CitAN1*	GGA AGG AAA CGA GAA GGA AAC A	R

Supplementary Table 7. Primers used for the cloning of *CsPH4* and *CsAN1* genes

primer	gene	sequence 5'-3'	orientation
6544	*CitPH4*	ATG AGG AAC CCA TCA ACA TCA C	F
6547	*CitPH4*	CTA CTC AAC GTG TTC ATC AAC C	R
6623	*CitAN1*	ATG GAT GCT CCG CCG CCG A	F
6596	*CitAN1*	TGA GTT AAT TGA CAT ACT GGG GT	R

Supplementary Table 8. Primers used for the amplification and qRT-PCR analysis of distinct mRNAs

primer	gene	sequence 5'-3'	orientation
7233	*CitACTIN11*	GGC ATT GCC GAT AGA ATG AGC A	F
7234	*CitACTIN11*	TCA TAC TCA GCC TTT GCA ATC CA	R
8934	*ANNEXIN2*	TGC TGA GCA ACT CCA CAA AGC	F
8935	*ANNEXIN2*	TCT CGA ATC AAC TTG CGC TGA G	R
8936	*ANKYRIN*	ATG AGG ATG AGT CAA TTG TTC ATC	F
8937	*ANKYRIN*	GCA ATG CTG TCC TTC CTT CTG	R
8938	*Ribosomal protein S10*	GAA ATC TGC AAG TAC CTC TTC C	F
8939	*Ribosomal protein S10*	CAT CAG CTT AAT CAC CTG CAG G	R
7666	*CitPH1*	CCA GAG ACA GAT GTG TCG TCA	F
7667	*CitPH1*	CAT GAT TGC ACG GTC CAT GGT	R
6530	*CitPH5*	GG AGC TCT GTT GAT GTG TGC A	F
6531	*CitPH5*	ATG AAC TTA ATG ACG TCC AAT GGT	R
7633	*CitMAC9F1*	GCA GAA ACG GAT TGA TGA AGA G	F
7634	*CitMAC9F1*	CAT CAG GGA CAA CTT CCT CAG	R
7664	*CitPH3*	CTG AGA TTC TTA GTG ATG GCT TC	F
7665	*CitPH3*	CAG ATG TCA CTG GAG TTG TGC	R
6545	*CitPH4*	GAT CTC ATT CTT CGC CTA CAT C	F
6546	*CitPH4*	CTT GGC TTA TCA GCT TCT TAC TC	R
7670	*CitAN11*	GGT CTC CTG CAC AGC CTG A	F
6615	*CitAN11*	AGT TAG CTA TCC CAA ACG AGC A	R
6577	*CitAN1 5'*	CGGT GAA TAC AGT CGG TAA AGG A	F
7575	*CitAN1 5'*	CTG CAC TGT TTT CCT AGT CTT GA	R
6579	*CitAN1 mid*	ACC GGT TAT GAT AGG TAG CAG T	F
6580	*CitAN1 mid*	GCT GCT TCT GAA GAA TGG TAG A	R
7598	*CitAN1 3'*	GAA CAA AGA AGG GCT GTT GCT	F
7599	*CitAN1 3'*	CTT TCT TCC CGT TTA CAT TGT CC	R
7807	*CitSO 5'*	CTT CTG GGA GAA AGC TTT TGA ATG	F
7808	*CitSO 5'*	CCA GAA CAA CAT TGA TAG GAA TGA	R

Supplementary Table 9. Primers used for the preparation of construct for transient transformation

primer	clone	sequence 5'-3'	orientation
6623	*Fso AN1 2.2kb full size cDNA*	ATG GAT GCT CCG CCG CCG A	F
6593	*Faris Sour AN1 2.2kb full size cDNA*	G AAA ATT TAA ATG CCA AAT GC	R
6623	*Faris sweet AN1 1.3kb truncated cDNA*	ATG GAT GCT CCG CCG CCG A	F
6595	*Faris sweet AN1 1.3kb truncated cDNA*	AGA AGC AGA GAA TCT CTG GCG GTG A	F
6572	*Faris Sour PH4 cDNA*	ATG AGG AAC CCA TCA ACA T	F
65a	*Faris Sour PH4 cDNA*	GGGGACCACTTTGTACAAGAAAG	R
6572	*Faris Sweet PH4 cDNA*	ATG AGG AAC CCA TCA ACA T	F
65a	*Faris Sweet PH4 cDNA*	GGGGACCACTTTGTACAAGAAAG	R
7326	*Faris Sour PH1 3.3kb promoter*	CT TCG GAT TCT GAA TCT T	F
7328	*Faris Sour PH1 3.3kb promoter*	TCT CTT CCT CTT CAG TTC TAG AC	R
7194	*Faris Sour PH5 3kb promoter*	GAG GTA CAT TGA CCT ACA	F
7195	*Faris Sour PH5 3kb promoter*	CAG ACG AAA GTC CTT GTC GAG	R

Supplementary references

1. Verweij, W. *et al.* Functionally similar WRKY proteins regulate vacuolar acidification in petunia and hair development in Arabidopsis. *Plant Cell* **28**, 786-803 (2016).
2. de Vetten, N., Quattrocchio, F., Mol, J. & Koes, R. The *an11* locus controlling flower pigmentation in petunia encodes a novel WD-repeat protein conserved in yeast, plants and animals. *Genes Dev.* **11**, 1422-1434 (1997).
3. Xu, Q. *et al.* The draft genome of sweet orange (*Citrus sinensis*). *Nat Genet* **45**, 59-66 (2013).
4. Quattrocchio, F. *et al.* PH4 of petunia is an R2R3-MYB protein that activates vacuolar acidification through interactions with Basic-Helix-Loop-Helix transcription factors of the anthocyanin pathway. *Plant Cell* **18**, 1274-1291 (2006).
