# Supplementary material for: Hyperacidification of Citrus fruits by a vacuolar proton-pumping P-ATPase complex
Source: Nat Commun. 2019 Feb 26;10:744. doi: 10.1038/s41467-019-08516-3 (PMC6391481; doi:10.1038/s41467-019-08516-3)
Supplement: Supplementary file 4 — Reporting Summary [file 41467_2019_8516_MOESM4_ESM.pdf]

nature research

Corresponding author(s): Ronald Koes and Francesca Quattrocchio

Reporting Summary

Nature Research wishes to improve the reproducibility of the work that we publish. This form provides structure for consistency and transparency in reporting. For further information on Nature Research policies, see Authors & Referees and the Editorial Policy Checklist.

Statistical parameters

When statistical analyses are reported, confirm that the following items are present in the relevant location (e.g. figure legend, table legend, main text, or Methods section).

n/a	Confirmed	
☐	☒	The exact sample size (n) for each experimental group/condition, given as a discrete number and unit of measurement
☐	☒	An indication of whether measurements were taken from distinct samples or whether the same sample was measured repeatedly
☒	☐	The statistical test(s) used AND whether they are one- or two-sided<br>*Only common tests should be described solely by name; describe more complex techniques in the Methods section.*
☒	☐	A description of all covariates tested
☒	☐	A description of any assumptions or corrections, such as tests of normality and adjustment for multiple comparisons
☐	☒	A full description of the statistics including central tendency (e.g. means) or other basic estimates (e.g. regression coefficient) AND variation (e.g. standard deviation) or associated estimates of uncertainty (e.g. confidence intervals)
☒	☐	For null hypothesis testing, the test statistic (e.g. F, t, r) with confidence intervals, effect sizes, degrees of freedom and P value noted<br>*Give P values as exact values whenever suitable.*
☒	☐	For Bayesian analysis, information on the choice of priors and Markov chain Monte Carlo settings
☒	☐	For hierarchical and complex designs, identification of the appropriate level for tests and full reporting of outcomes
☒	☐	Estimates of effect sizes (e.g. Cohen's d, Pearson's r), indicating how they were calculated
☐	☒	Clearly defined error bars<br>*State explicitly what error bars represent (e.g. SD, SE, CI)*

Our web collection on statistics for biologists may be useful.

Software and code

Policy information about availability of computer code

Data collection	no software was used for data collection
Data analysis	1) Sequence alignments were made with Clustal-Omega or MUSCLE and adjusted manually with Aliview. Phylogenetic trees were generated with PHYML and visualized with Treeview, sequence alignments were colored with BOXSHADE, all using online tools, as indicated in Methods<br>2) Real time PCR data were analyzed QuantStudio™ Design and Analysis Software v1.4.3.and further elaborated in Microsoft Excell 2011 to generate graphs<br>3) Figures were assembled with Adobe Photoshop and Illustrator (CS6).

For manuscripts utilizing custom algorithms or software that are central to the research but not yet described in published literature, software must be made available to editors/reviewers upon request. We strongly encourage code deposition in a community repository (e.g. GitHub). See the Nature Research guidelines for submitting code & software for further information.

Data

Policy information about availability of data

All manuscripts must include a data availability statement. This statement should provide the following information, where applicable:

- Accession codes, unique identifiers, or web links for publicly available datasets
- A list of figures that have associated raw data
- A description of any restrictions on data availability

Identifiers for genes in distinct Citrus species and varieties can be found in Supplementary Table 2. Sequence data on alleles analysed in this study have been deposited in NCBI-Genbank and are accessible under accession numbers MH843936-MH843962 (cDNAs of CitSO and CitPH3), MH885854-MH885946 (genomic DNAs of CitAN1, CitPH4, CitPH3, CitFAR-like, CitTFIIH-like) and MH898434-MH898465 (cDNAs of CitAN1, CitPH4, CitAN11, and CitPH3).

Field-specific reporting

Please select the best fit for your research. If you are not sure, read the appropriate sections before making your selection.

☒ Life sciences ☐ Behavioural & social sciences ☐ Ecological, evolutionary & environmental sciences

For a reference copy of the document with all sections, see nature.com/authors/policies/ReportingSummary-flat.pdf

Life sciences study design

All studies must disclose on these points even when the disclosure is negative.

Sample size	For all analyses at least two technical replicates (mRNA measurements on same RNA samples) were used. For many, but not all, varieties we used two or more biological replicates (i.e. measurements on different fruits), as it was more informative to analyse X different fruits from X different varieties with either high or low acidity than to analyze X replicates from a single variety.
Data exclusions	1.Expression analysis of several citrus genes (e.g. CitJAF13, Ruby) were excluded from the data, as they proved not informative.<br>2. Expression of several genes was monitored by RT-PCR or qPCR with different primers combinations. As all primer combinations produced essentially similar results (except for some CitAN1 primer combinations on varieties harbouring alleles in which the 3' end was deleted) , for most genes only results with a single primer set are shown/mentioned.<br>3.qPCR replicates made by different RNA extractions of the same fruits – which are somewhere between true biological and technical replicates – were always consistent, but have not been included in the shown data.
Replication	All attempts at replication were successful. Data on pH titratable acid content and soluble solid content (Brix) presented in this study, were in line with (unpublished) data obtained on the same trees/varieties over multiple years by the team at UC-Riverside
Randomization	No methods for randomization were used to determine experimental groups
Blinding	Researchers were not blinded to group allocation

Reporting for specific materials, systems and methods

Materials & experimental systems

n/a	Involved in the study
☐	☒ Unique biological materials
☒	☐ Antibodies
☒	☐ Eukaryotic cell lines
☒	☐ Palaeontology
☒	☐ Animals and other organisms
☒	☐ Human research participants

Methods

n/a	Involved in the study
☒	☐ ChIP-seq
☒	☐ Flow cytometry
☒	☐ MRI-based neuroimaging

Unique biological materials

Policy information about availability of materials

Obtaining unique materials	Gene constructs and petunia line(s) are freely available on request. Fruits and of citrus accessions from the Citrus Variety

April 2018

Obtaining unique materials	Collection and Citrus Experiment Station at Riverside, California are available on request from M.L.R and C.T.F. Availability of fruits or tissue is dependent on growing season and on the recipient providing an appropriate import permit if required.
